# Same-day discharge after early mobilisation and increased frequency of physiotherapy following hip and knee arthroplasty

**DOI:** 10.4102/sajp.v78i1.1755

**Published:** 2022-05-31

**Authors:** Retha-Mari Prinsloo, Monique M. Keller

**Affiliations:** 1 Department of Physiotherapy, Faculty of Health Science, University of the Witwatersrand, Parktown, South Africa

**Keywords:** length of stay, early mobilisation, physiotherapy, arthroplasty, replacement, hip, knee

## Abstract

**Background:**

Advanced rehabilitation pathway (ARP) after hip and knee arthroplasties is popular globally and is gaining ground in South Africa (SA). A multidisciplinary team in Rustenburg, SA, has implemented an ARP with the first same-day discharge (SDD) from hospital. The lack of evidence of physiotherapy protocols within an ARP determined our study.

**Objectives:**

Determine and compare hospital length of stay (LOS) (hours), patient satisfaction (Western Ontario and McMaster Universities Osteoarthritis Index (WOMAC)), patient safety (30-day re-admission) and cost between the two cohorts.

**Method:**

A quantitative prospective patient (treatment) group receiving early mobilisation with increased frequency of physiotherapy on post-operative day zero (POD0) was compared to a conservatively managed retrospective historical (control) group following post-operative elective hip and knee arthroplasties.

**Results:**

Results for the prospective group which were significantly improved relative to the retrospective group included decreased LOS (median 7.650, *p* < 0.001), less pain at 6 weeks (mean 16.20, standard deviation [SD] = 2.673, *p* < 0.001), less stiffness (mean 5.82, SD = 1.214, *p* = 0.007), higher function (mean 54.87, SD = 8.544, *p* < 0.001), lower hospital cost (mean R43 340, *p* < 0.001) and physiotherapy cost (mean R1069, *p* < 0.001), and total costs compared to the retrospective group (mean R117 062, *p* < 0.001).

**Conclusion:**

Safe and cost-effective SDD is possible in an ARP with earlier mobilisation and increased frequency of physiotherapy on POD0.

**Clinical implications:**

Achieving safe SDD after hip and knee arthroplasty surgeries saved costs and improved patient satisfaction, with a decrease in LOS being beneficial for medical funders and stakeholders including government aiming to implement National Health Insurance (NHI) in the future.

## Introduction

Osteoarthritis (OA) is a chronic degenerative joint disease that can affect any joint (Contartese et al. 2020). Osteoarthritis affects women more than men and can decrease function and independence, thus reducing quality of life (Hawker 2019). Individuals living with symptomatic hip and/or knee OA number an estimated 242 million (3.8% of the world population) people worldwide (Carlesso et al. 2016). Musculoskeletal and orthopaedic diseases such as OA are on the rise in South Africa (SA) because of lifestyle changes, obesity, increased life expectancy, trauma and the high incidence of human immunodeficiency virus (HIV) (Plenge et al. 2018).

Hip and knee arthroplasty have been effective as interventions for end-stage OA since the 1970s, treating pain, stiffness, decreased function and quality of life where conservative treatment methods failed (Gademan et al. 2016; Sculco & Pagnano 2015). In the United States of America (USA), the demand for joint arthroplasties is predicted to increase by 174% for hips and 673% for knees by 2030 from the demand in 2007 (Kurtz et al. 2007). According to Rupp et al. (2016), it is predicted that joint arthroplasties will increase by 23% for hips and 45% for knees in Germany between 2016 and 2040.

The increased demand for hip and knee arthroplasties creates an enormous financial strain on the South African health care system. Elective surgeries being postponed during the coronavirus disease 2019 (COVID-19) pandemic to prioritise hospital resources and staff for the care of COVID-19 patients led to extended waiting times, further delaying elective hip and knee arthroplasties (Anderson et al. 2021; The Lancet Rheumatology 2021).

Multidisciplinary advanced rehabilitation pathways (ARPs) have been introduced globally to deliver good-quality health care in a cost effective, safe manner while improving patient satisfaction and outcomes in comparison to more conservative protocols (Immelman, De Vos & Venter 2018; Plenge et al. 2018; Riemer et al. 2017; Robertson et al. 2015; Tayrose et al. 2013). There are several ways to decrease the cost of arthroplasties, one being pre-operative education and early mobilisation that decreases hospital length of stay (LOS) and post-operative complications (Riemer et al. 2017). These ARP pathways focus on standardised care, including patient education, multi-modal opioid-sparing pain control, thromboprophylaxis, restricting blood loss during surgery and early mobilisation (Lazic et al. 2018; Riemer et al. 2017).

Prolonged hospital stay is associated with increased mortality and morbidity following joint arthroplasty (Lazic et al. 2018; Maempel et al. 2016; McCulloch et al. 2017). By safely implementing ARP and gradually decreasing hospital LOS, these pathways have led to same-day discharge (SDD) joint arthroplasties in Europe and the USA (Yates et al. 2018). Same-day discharge means patients are discharged on the same calendar day as the surgery (McCulloch et al. 2017). Jean-Yves and Gisonni (2022) suggest that reluctance to use ambulatory or SDD is based on concerns of increased complication rates. A number of studies have found that there is no significant increase in complication rate with SDD when compared to longer LOS (Bovonratwet et al. 2020; Jean-Yves & Gisonni 2022; Kelmer, Turcotte & King 2021). Common complications identified in these studies are stiffness, delayed wound healing, infections, dislocation and fractures, thromboembolic complications, swelling and pain. Continuous improvement of pre-operative education on home medication and oedema management will further decrease the risk of complications (Kelmer et al. 2021)

As part of the multidisciplinary team, physiotherapists play an essential part of the ARP with patient education and early mobilisation. Pre-operative education decreases patient expectations, improves patient knowledge, improves knee flexion range of movement and improves post-operative performance specifically exercise and functional activities (Jordan et al. 2014). The combination of education and early mobilisation decreases hospital LOS and the cost of knee arthroplasty (Jordan et al. 2014). Early mobilisation post-operative day zero (POD0) decreases hospital LOS significantly (Lazic et al. 2018; Masaracchio et al. 2017; McCulloch et al. 2017; Riemer et al. 2017; Tayrose et al. 2013; Yakkanti et al. 2019) and also decreases post-operative complications like deep venous thrombosis, infections related to the prosthesis and postural hypotension (Chen et al. 2012; Dossett & Chesser 2017). In a systematic review and meta-analysis, Masaracchia et al. (2017) found that early initiation of rehabilitation 1 – 4 h post-operative (Raphael, Jaeger & Van Vlymen 2011; Tayrose et al. 2013) on the day of surgery decreases the LOS without increasing adverse events or readmission rate in patients following joint arthroplasty surgery.

Several factors have been identified in studies that can make early mobilisation easier. Opioid-sparing pain control decreases dizziness, nausea, orthostatic hypotension and sleepiness, and the absence of surgical drains and urine catheters make mobilisation easier and improve patient independence (Lazic et al. 2018; Sharma, Palekar & Tanna 2016). Using a tourniquet sparingly during surgery and at appropriate pressures is associated with decreased post-operative pain, and the combination of general anaesthesia and local infiltration anaesthesia has fewer post-operative complications and allows for early mobilisation because the motor function is preserved (Marques et al. 2014). Quick patient transfers from the recovery room to the ward and the availability of physiotherapists even for patients returning from theatre late in the day, allows for early mobilisation of patients and therefore may decrease LOS (Guerra, Singh & Taylor 2015).

Despite arthroplasty being a cost-effective way of treating OA and the progress made in decreasing hospital LOS, studies have indicated a 10% for total hip arthroplasty (THA) and 20% for total knee arthroplasty (TKA) patient dissatisfaction with outcomes (Gill & McBurney 2013; Gunaratne et al. 2017). Follow-up periods for studies include 3 months up to 3.5 years, with 1-year post-operative being the most common follow-up period following arthroplasty surgery (Gunaratne et al. 2017). Factors such as pre-operative patient expectations and post-operative pain, stiffness, function and complications influence patient satisfaction (Gill & McBurney 2013; Walker et al. 2018). Patients expect a decrease in pain and stiffness and improvement in function and quality of life following an arthroplasty (Gunaratne et al. 2017; Thambiah et al. 2015).

Since implementing an ARP with early mobilisation POD0 at a private hospital in Rustenburg, SA, the average LOS decreased from 3.5 days to 23 h. Physiotherapy as part of the multidisciplinary ARP plays a vital role, and with a lack of studies in this area on best evidence practice for arthroplasty management in SA, the aim of our study was to investigate the effect of early mobilisation and increased frequency of physiotherapy on POD0 on patient outcomes following elective hip and knee arthroplasty in a private hospital in SA.

## Method

Our prospective cohort study included a purposive convenient, selected sample of patients (*n* = 60). With the margin for error set on 0.05 and power of 95%, we calculated that *n* = 53 patients were required for our study to compare the two cohorts. The ARP guided the management following hip and knee arthroplasty surgery. The prospective cohort was compared to a retrospective control group (*n* = 60), managed with a more conservative protocol. The sample size was determined by the total number of patients who underwent either a total hip or knee arthroplasty in the retrospective control group year at the private hospital performed by the orthopaedic surgeon who implemented the ARP and consented to our study.

The setting and multidisciplinary team for both the prospective treatment group and the retrospective control group were the same. The multidisciplinary team for all surgeries consisted of an orthopaedic surgeon, anaesthetist, physiotherapist and nursing staff. The protocols stayed consistent throughout our study.

The physiotherapy protocol for the retrospective control group included early mobilisation with exercises 3 hours post-intervention on POD0. The prospective cohort physiotherapy protocol included early mobilisation with exercises 1 – 3 hours post-operative on POD0 and a second mobilisation with an exercise session 1 to 2 hours later. The physiotherapy protocol included a pre-intervention physiotherapy education session, assessments of outcomes, the post-intervention session/s, assessment, and criteria before discharge. For the detailed protocol the proposal article may be read (Prinsloo & Keller 2021).

All consecutive elective hip and knee arthroplasty patients who were cleared pre-operatively by the anaesthetist or general physician (depending on co-morbidities) as per the ARP protocol were included. Participants received information regarding the purpose of our study, and informed consent was obtained. Patients excluded were trauma-related arthroplasty, bilateral arthroplasty, revision surgery and cognitive deficiencies. Patients with conditions affecting their balance or poor balance observed during the education sessions by either the orthopaedic surgeon or physiotherapist were also excluded. Demographically the prospective and retrospective groups were matched according to age, gender, body mass index (BMI) and the type of arthroplasty.

Patients in both groups (retrospective and prospective) received the same multi-disciplinary ARP management and protocol at the Medicare Private Hospital in Rustenburg, the difference being the time before the first mobilisation and the frequency of physiotherapy treatment on POD0. The previous more conservative protocol as per the retrospective group included a pre-operative education session in the hospital and patients mobilising once on POD0, 3 hours post-operative. With the new protocol, patients received an education session the week before surgery, mobilising 1 to 3 hours post-operative (Raphael et al. 2011; Tayrose et al. 2013) and then again for a second time 1 to 2 hours after the first session. Standardised verbal instructions and procedures were used in a standardised environment when collecting data to ensure and improve reliability with the outcome measures listed below.

### Outcome measures

Length of stay is often used as an outcome measure following hip and knee arthroplasty and measured in mean number of days. Length of stay was measured in hours to be more accurate and to detect subtle changes in LOS more effectively (McCulloch et al. 2017). Length of stay was calculated from the time the patient went to theatre until discharge.

The Western Ontario and McMaster Universities Osteoarthritis Index (WOMAC) is a frequently used, valid and reliable outcome measure for patients following hip and knee arthroplasty surgery measuring the total score and subscores for pain, function and stiffness (Collins et al. 2011; Giesinger et al. 2015). The score is calculated according to an ordinal scale of 0–4. However, recent studies (Walker et al. 2018) have used a reverse scale from 4 to 0 (none, mild, moderate, severe and extreme), with a total score of 100 being the best possible outcome and 0 being the worst possible outcome. We used the reversed scale to score.

Patient satisfaction was measured as suggested by the International Society of Arthroplasty Registries Patient-reported Outcome Measures (PROMs) Working Group, using a one-item satisfaction outcome (Rolfson et al. 2016). A single question, ‘How satisfied are you with your hip/knee arthroplasty?’, was posed to patients on a five-point Likert scale, with 1 extremely dissatisfied, 2 dissatisfied, 3 neutral, 4 satisfied and 5 extremely satisfied (Thambiah et al. 2015). Participants were then grouped as either satisfied (4–5) or dissatisfied (1–3).

Patient safety was measured by documenting any adverse events or readmissions within the first 30 days following surgery. Lastly the direct cost of hospital LOS was compared between the prospective and retrospective cohorts. Hospital (including theatre), orthopaedic surgeon, anaesthetist, physiotherapy and assistive device costs were considered and included in the simple cost comparison.

### Statistical analysis

Data analysis was performed using the IBM SPSS 27 (International Business Machine Statistical Package for the Social Sciences) version 27 and *p* < 0.05 was considered statistically significant. Descriptive statistics, namely frequencies and percentages for categorical data and means and standard deviations (SD), or medians and percentiles for numerical data, were calculated. Quantitative outcome variables were tested for normality using the Shapiro-Wilk’s test to assess whether parametric tests were appropriate or not. If data were found to be normally distributed, parametric tests were used, and if not normal, then non-parametric tests were used. Safety data were collected as binary data. Demographics were compared between the treatment groups using *t*-tests for quantitative demographic variables such as age, BMI and chi-square tests in the case of categorical demographics.

Length of stay was presented using median and inter-quartile ranges and for comparison between the two treatments groups using a non-parametric Mann-Whitney test because this variable was not normally distributed. Western Ontario and McMaster Universities Osteoarthritis Index scores were normally distributed and thus summarised using mean and SD and a comparison between the two treatment groups using *t*-tests. Repeated measures Analysis of Variance (ANOVA) tests of the effect of time were used in the treatment group to assess the significance of the change in scores over the three time points using the Wilk’s lambda statistic. This is the appropriate test for comparison of three paired means in normally distributed data. The occurrence of adverse events was compared between the two treatment groups using Fisher’s exact test. Cost of LOS data was summarised using median and inter-quartile ranges and the two treatment groups compared using non-parametric Mann-Whitney tests.

### Ethical considerations

Our trial is registered with the Pan African Trial Registry (trial number: PACTR202103637993156). Ethical clearance was obtained from the University of the Witwatersrand Human Research Ethics (Medical) Committee (clearance number: M200576), and consent from the orthopaedic surgeon and the manager at the private hospital in Rustenburg where data collection took place. Permission for using data from the hospital records for data collection in the main prospective and retrospective cohort group was included. Information regarding the research and participation was provided to prospective participants and they all signed an informed consent form.

## Results

### Demographics

There was no difference in the mean age (*p* = 0.217) or BMI (*p* = 0.903) between the groups as summarised in Table 1.

**TABLE 1:** Patient demographics as per age and body mass index (*n* = 120).

Variables	Group		*p* †
	Retrospective control	Prospective treatment	
**Age**			0.217
Mean	62.00	59.00	-
Standard deviation	10.00	11.00	-
**BMI**			0.903
Mean	31.89	31.74	-
Standard deviation	6.39	6.84	-

BMI, body mass index.

† , Independent samples *t*-test were used to get the *p*-value.

The demographics of the study sample are shown per group according to gender and arthroplasty type (Table 2). There was no difference between the retrospective and prospective groups.

**TABLE 2:** Patient demographics as per gender and arthroplasty type (*n* = 120).

Variables	Group				*p* †
	Retrospective control		Prospective treatment		
	*n*	%	*n*	%	
**Gender**					0.361
Male	34	56.7	29	48.3	-
Female	26	43.3	31	51.7	-
Total	60	100.0	60	100.0	-
**Type**					0.674
TKA	30	50.0	33	55.0	-
PNA	15	25.0	11	18.3	-
THA	15	25.0	16	26.7	-
Total	60	100.0	60	100.0	-

TKA, Total knee arthroplasty; PKA, partial knee arthroplasty; THA, total hip arthroplasty.

† , Chi square test was used to get the *p*-value.

There was a statistically significant difference in LOS between the two groups (*p* < 0.001). The median hours were much higher in the retrospective group (median = 43.15) than in the prospective group (median = 7.65), as shown in Table 3.

**TABLE 3:** Comparison of hospital length of stay in hours between groups (medians and inter-quartile ranges) (*n* = 120).

Variables	Group		*p* †
	Retrospective control	Prospective treatment	
**LOS hours**			< 0.001
Median	43.150	7.650	-
Percentile 25	27.133	6.292	-
Percentile 75	49.433	21.249	-

LOS, Length of stay.

† , Mann-Whitney test was used to get the *p*-value.

Data were normally distributed for the WOMAC subscale and there were no differences pre-operatively between the two groups, but at 6 weeks, each of the WOMAC scales and the total scores were statistically significantly different between the two groups (Table 4) in favour of the prospective group.

**TABLE 4:** Comparison of patient reported outcome Western Ontario and McMaster Universities Osteoarthritis index between groups (*n* = 120).

Variable	Group	*n*	Mean	Standard deviation	Standard error mean	95%	Confidence interval (CI)	*p* †
Pre-operative pain	-	-	-	-	-	−1.034	1.700	0.630
	Retrospective control	60	8.18	3.661	0.473	-	-	-
	Prospective treatment	60	7.85	3.896	0.503	-	-	-
Pre-operative stiffness	-	-	-	-	-	−1.099	0.299	0.259
	Retrospective control	60	2.83	1.758	0.227	-	-	-
	Prospective treatment	60	3.23	2.094	0.270	-	-	-
Pre-operative function	-	-	-	-	-	−2.811	5.611	0.512
	Retrospective control	60	27.00	11.102	1.433	-	-	-
	Prospective treatment	60	25.60	12.166	1.571	-	-	-
Total WOMAC score pre-operative	-	-	-	-	-	−4.077	7.277	0.578
	Retrospective control	60	39.67	15.454	1.995	-	-	-
	Prospective treatment	60	37.93	17.292	2.232	-	-	-
6/52 pain	-	-	-	-	-	−3.413	−1.554	< 0.001
	Retrospective control	60	13.72	2.464	0.318	-	-	-
	Prospective treatment	60	16.20	2.673	0.345	-	-	-
6/52 stiffness	-	-	-	-	-	−1.033	−0.167	0.007
	Retrospective control	60	5.22	1.180	0.152	-	-	-
	Prospective treatment	60	5.82	1.214	0.157	-	-	-
6/52 function	-	-	-	-	-	−7.803	−2.564	< 0.001
	Retrospective control	60	49.68	5.655	0.730	-	-	-
	Prospective treatment	60	54.87	8.544	1.103	-	-	-
Total WOMAC score at 6 weeks	-	-	-	-	-	−12.622	−5.043	< 0.001
	Retrospective control	60	71.58	8.280	1.069	-	-	-
	Prospective treatment	60	80.13	12.022	1.552	-	-	-

WOMAC, Western Ontario and McMaster Universities Osteoarthritis Index.

† , *t*-test were used to get the *p*-value.

### Patient satisfaction up to three months in the treatment group

**FIGURE 1:**
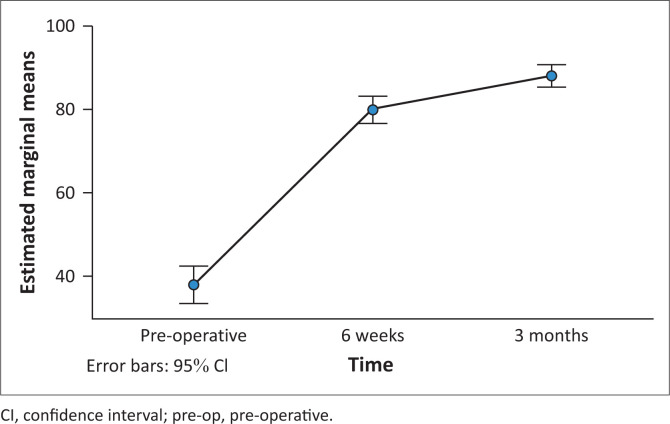
Western Ontario and McMaster Universities Osteoarthritis index mean total score over time with 95% confidence interval in treatment group.

Pain, stiffness, function and total WOMAC scores increased statistically significantly over time in the treatment group (*p* < 0.001). Figure 1 shows the means of the total score over time at 95% confidence intervals. The highest change was between the pre-operative and 6-week periods.

A single question, ‘How satisfied are you with your hip/knee arthroplasty?’, was posed to patients 3 months post-operative on a five-point Likert scale, with1 extremely dissatisfied, 2 dissatisfied, 3 neutral, 4 satisfied and 5 extremely satisfied (Rolfson et al. 2016; Thambiah et al. 2015). Participants’ responses were then grouped as either satisfied (4–5) or dissatisfied (1–3); 98% of participants stated that they were satisfied, and only 2% (one patient) reported dissatisfaction, with one patient stating no relief in pain.

The results regarding the adverse events indicated that there were six readmissions. In the control group, 6.7% (*n* = 4) participants were readmitted, and in the treatment group 3.3% (*n* = 2) participants were readmitted. The difference was small and not statistically significant (*p* = 0.679 – Fisher’s exact two-sided test), as shown in Table 5. The estimated difference between the groups in terms of readmission was 3.4% (95% CI: 4.3% – 11.18%).

**TABLE 5:** Comparison of patient safety 30-day readmission rate between the two groups.

Variables	Group				Total	
	Retrospective control		Prospective treatment			
	*n*	%	*n*	%	*n*	%
**Readmission**						
0	56.0	93.3	58.0	96.7	114.0	95.0
1	4.0	6.7	2.0	3.3	6.0	5.0

**Total**	**60.0**	**100.0**	**60.0**	**100.0**	**120.0**	**100.0**

Fisher’s exact two-sided test.

There was a statistically significant difference in the hospital costs (*p* < 0.001) between the groups, with the retrospective group having higher hospital costs than the prospective group. The findings were similar for physiotherapy costs (*p* < 0.001) and total costs (*p* < 0.001). However, prosthesis costs were significantly higher in the prospective group (*p* = 0.004) (Table 6).

**TABLE 6:** Simple in-hospital cost comparison between groups (median and inter-quartile range).

Variables	Group		*p* †
	Retrospective control	Prospective treatment	
**Hospital cost**			< 0.001
Median	R 53 703	R 43 340	-
Percentile 25	R 48 355	R 37 072	-
Percentile 75	R 58 937	R 48 188	-
**Orthopaedic surgeon**			0.415
Median	R 24 910	R 24 205	-
Percentile 25	R 20 000	R 22 666	-
Percentile 75	R 33 000	R 28 250	-
**Anaesthetist**			0.811
Median	R 8500	R 8034	-
Percentile 25	R 6292	R 6244	-
Percentile 75	R 10 467	R 8800	-
**Physiotherapy treatment**			< 0.001
Median	R 1923	R 1069	-
Percentile 25	R 1600	R 832	-
Percentile 75	R 2327	R 1248	-
**Prosthesis**			0.004
Median	R 36 700	R 38 350	-
Percentile 25	R 35 435	R 38 000	-
Percentile 75	R 42 171	R 39 603	-
**Assistive devices**			0.416
Median	R 881	R 673	-
Percentile 25	R 521	R 485	-
Percentile 75	R 1318	R 1314	-
**Total cost**			< 0.001
Median	R 128 416	R 117 062	-
Percentile 25	R 124 367	R 111 636	-
Percentile 75	R 137 157	R 125 862	-

† , Mann-Whitney test was used to get the *p*-value.

The average number of physiotherapy treatment sessions in the retrospective group was five, and two in the prospective group, with a statistically significant difference between the groups (*p* < 0.001).

## Discussion

The primary outcome measure LOS was measured in hours as suggested by McCulloch et al. (2017) to be more accurate in documentation, detect more minor changes and decrease outlier distortion. With LOS below 24 hours, day-case arthroplasty could now be considered. Since implementing earlier mobilisation within 1 to 3 hours post-operative and increasing the frequency of treatment adding a second session 1 to 2 hours later, our results indicated a statistically significant decrease in LOS with the prospective treatment group at 7.65 hours compared to the retrospective control group at 43.15 hours. There was no difference in LOS between the different types of arthroplasties (*p* = 0.283). Supporting our study, numerous studies have found that early mobilisation on POD0 following joint arthroplasty is associated with a significant decrease in LOS without an increase in adverse events (Masaracchio et al. 2017). Auyong et al. (2021) showed LOS to be reduced by 20 h, and Yakkanti et al. (2019) found a significant decrease (*p* = 0.002) in LOS in the group of patients that was out of the theatre by 17:00 and mobilising POD0 versus the group mobilising POD1. A meta-analysis of five randomised control trials showed a decrease in LOS by 1.8 days (43.2 h) after early mobilisation on POD0 following hip and knee arthroplasty (Guerra et al. 2015). Reasons for delayed discharge were identified as a lack of physiotherapy resources, delayed transfers from the recovery room back to the ward, using motor nerve blocks affecting the lower limb muscle motor control and low patient motivation levels (Guerra et al. 2015). A multidisciplinary approach is needed to optimise patients pre-operatively, using multi-modal opioid-sparing analgesia and early mobilisation to ensure early discharge (Yakkanti et al. 2019). In our study all patients received a combination of general anaesthesia and adductor canal block preserving muscle control, with swift transfer to the ward and mobilisation after 1 to 3 hours. Mobilisation was performed with the assistance of the physiotherapist on call to accommodate the increased demand to ensure early mobilisation and discharge. Patient motivation was optimised, and anxiety decreased in the pre-operative education session provided by the physiotherapist.

Jenkins et al. (2019) encouraged patients to avoid knee flexion following knee arthroplasty because the authors thought it would increase swelling and pain, and delay mobilisation and discharge. By mobilising early on POD0, the LOS decreased and SDD was possible in 39% of patients, and 38% of patients were discharged on POD1. Our patients started knee flexion exercises immediately after their TKA up to 90°, with no delay in mobilisation found in 61.66% (*n* = 37) patients with SDD and 33.33% (*n* = 20) discharged POD1. In contrast to Jenkins et al. (2019), we did not find early knee flexion to delay mobilisation or to delay discharge.

Lenssen et al. (2006) found that increasing the frequency of treatment on POD0 from one to two sessions did not make a significant difference in LOS, pain, function or knee ROM. This might be because their study protocol was not part of a multi-disciplinary ARP and no pre-operative education sessions were performed. In contrast to this, we experienced that those patients who received only one session on POD0 in the retrospective control group tended not to mobilise again until they saw the physiotherapist the next morning. Firstly, patients seemed to not have the confidence to mobilise alone and, secondly, nursing staff were reluctant to assist patients to mobilise to the toilet during the night and would instead use bedpans owing to fearing that patients may fall.

In our prospective group, patients received a second session 1 to 2 hours after the first session and gained confidence in their own functional ability and the belief that they would cope at home. A large percentage of patients could progress to climbing stairs and walk independently out of the hospital after discharge with their mobility aid. Patients who only mobilised the following morning for the second time showed readiness for discharge only the day following the surgery. From this, it seems that two physiotherapy sessions in the hospital on the day of the surgery led to patients being ready for discharge and achieving discharge criteria sooner compared to only one physiotherapy session. The number of physiotherapy sessions needed to make patients prepared for discharge is currently a relevant topic. Certain funders will allow patients three in-hospital sessions but restricting providers to only one session per day. So, if the average number of sessions needed for patients to be discharge ready is two sessions, patients would need to wait till the next day to receive the second session, thereby increasing hospital cost versus the cost of the second physiotherapy session.

Rules like these by funders should be reviewed not to hinder early discharge and SDD. The role of physiotherapists in SDD should be recognised by funders and providers should be compensated accordingly. In our prospective treatment group, 66% of patients achieved SDD through early mobilisation with increased frequency of physiotherapy. It is therefore recommended that if teams wish to progress to next day and eventually SDD, there should be a physiotherapist on call to mobilise patients out of bed on POD0 even when they return late from theatre. We also recommend that patients for hip and knee arthroplasy are first on the theatre list to allow enough time to reach functional goals and discharge criteria set at their pre-operative education session.

Three months post-operative when posed a single question, ‘how satisfied are you with your hip/knee arthroplasty?’, 98% (*n* = 59) of participants indicated on a five-point Likert scale that they were satisfied, and only one patient (2%) was dissatisfied, with one patient indicating no relief in pain. This is similar to the 98% of patients being extremely satisfied in a study by Riemer et al. (2017) 3 months post-operatively. Only one patient felt stressed and hurried by early discharge associated with the rapid recovery pathway. Walker et al. (2018) in their study found that 89.7% of patients were satisfied and only 10.3% dissatisfied following TKA out of 2589 patients, indicating that the WOMAC post-operative score can be reliably used by the health care professional to classify patients’ satisfaction following arthroplasty as excellent, good, fair and poor at 1-year post-operatively as it is a measure of pain, function and stiffness (Collins et al. 2011; Giesinger et al. 2015).

We used the reversed scoring scale as suggested by Walker et al. (2021). No difference was found in the pre-operative WOMAC scores between the two groups. At 6 weeks post-operative, however, there was a significant difference between the two groups in favour of the prospective treatment group (*p* < 0.001), indicating that the prospective group had favourable short-term outcomes in terms of decreased pain and stiffness and improved function. This could be because of patients becoming independent functionally quicker with earlier and increased frequency of physiotherapy treatment POD0 and gaining confidence that they are able to cope on their own at home. Because there were no WOMAC scores available 3 months post-operatively for the retrospective group, this comparison was not possible. The mean total WOMAC score for the prospective group pre-operatively was 37.93 and at 3 months post-operatively it was 87.34. Riemer et al. (2017) had a mean total WOMAC score pre-operatively of 35 and 85 at 3 months post-operative after implementing a rapid recovery protocol and early mobilisation on POD0 6 h after surgery. The frequency of treatment was also increased with mobilisation done two to three times per day. For pain, stiffness and function scores, the prospective treatment group showed a statistically significant increase with less pain and stiffness, and higher function, as compared to the retrospective group. The greatest increase was seen between the pre-operative and 6 weeks post-operative participants’ scores (*p* < 0.001). Thambiah et al. (2015) also found that patients with increased WOMAC total and function scores were more satisfied. However, post-operative pain and stiffness were not statistically significant for patient satisfaction in their study.

To evaluate the safety of implementing an ARP with early mobilisation we compared the 30-day readmission rate or rate of adverse events between the two groups. In the retrospective group, 6.7% of patients were readmitted compared to 3.3% in the prospective group. Thus, the difference was small and statistically insignificant (*p* = 0.679). Several studies found that earlier mobilisation with increased frequency of physiotherapy on POD0 as part of an ARP can be implemented safely without an increase in adverse events (Krause et al. 2018; Riemer et al. 2017; Thompson et al. 2021; Yakkanti et al. 2019). The reasons for readmission in our study were because of a family member of a patient with a total hip replacement being concerned about excessive swelling around the thigh area. The patient was admitted by the family’s general practitioner without consulting the orthopaedic surgeon. This highlights the importance of educating patients and family members and/or caregivers on expectations following arthroplasty. Another patient was admitted with COVID-19, and this could not be linked to the patient’s hospital stay during the arthroplasty.

In a simple cost comparison of direct in-hospital cost between the two groups, we found a statistical difference, with higher costs incurred in the retrospective control group (*p* < 0.001). The total cost and cost of physiotherapy showed a statistically significant difference between the groups, with the retrospective group having higher costs (*p* < 0.001). The cost of physiotherapy decreased by 44.4% in the prospective group, and the reason for this is the decreased LOS and the average number of physiotherapy sessions decreasing from five sessions in the retrospective group to two sessions in the prospective group. There was no significant difference in the orthopaedic surgeon, anaesthetist or assistive device cost per arthroplasty. Prosthesis costs, however, were significantly higher in the prospective group (*p* = 0.004). This might also be because of more total knee arthroplasties than partial knee arthroplasties in the prospective group. Several studies found early mobilisation associated with decreased LOS and total hospital cost. Schultz, Segovia and Castillo (2019) found that early mobilisation decreased LOS from 3.4 days to 1.6 days (*p* < 0.001), decreasing hospital cost by 24.7% while also decreasing post-operative complications. Similarly, Pelt et al. (2017) found that by changing physiotherapy shifts having a physiotherapist on call after-hours for patients returning late from theatre led to more patients mobilising early on POD0 and a median cost saving of 28% was achieved in patients following total joint arthroplasty. There is a lack of studies to determine the cost saving in physiotherapy fees following early mobilisation as part of an ARP. Thompson et al. (2021) found in a systematic review of 13 manuscripts and 3370 patients of day-case total knee arthroplasties that both the patient and health care system benefit from decreased LOS with decreased cost, improved patient outcome in terms of function, decreased post-operative complications and 30-day readmission rate.

A limitation of our study is the retrospective nature of the control group. Because of the nature of the ARP, it was not possible to have a prospective control group because the orthopaedic surgeon no longer makes use of the old protocol. Another limitation was that WOMAC scores were not available for the retrospective control group for comparisons to be made at 3 months post-operatively. A suggestion for a future study is to compare outcomes at 6 weeks, 3 months and 1-year post-operative. A strength of our study is that, to our knowledge, it is the first study following the implementation of a detailed and documented physiotherapy protocol for hip and knee arthroplasty in an ARP. It is also the first study on early mobilisation and frequency of physiotherapy in SA, with results leading to SDD. A further strength of this study is that it included a cost comparison, augmenting the lack of studies in this field.

## Conclusion

South Africa is in the position to use the latest protocols to ensure individuals waiting for hip and knee arthroplasty surgeries that have been delayed because of COVID-19 receive earlier, safe and more cost-effective management. Reaching SDD requires a multidisciplinary approach. We demonstrated that SDD is possible in SA in patients following hip and knee arthroplasty by implementing a multidisciplinary ARP. Physiotherapy with patient education, early mobilisation and increased frequency of treatment on POD0 and as part of this ARP led to improved patient-reported outcomes and satisfaction in a safe and cost-effective manner.
